# Supplementary material for: Integrated analysis of the aqueous humor microbiome and lens capsule transcriptome in high myopia cataract: a pilot study
Source: Front Med (Lausanne). 2026 Jun 16;13:1845205. doi: 10.3389/fmed.2026.1845205 (PMC13314463; doi:10.3389/fmed.2026.1845205)
Supplement: Supplementary file 3 [file Data_Sheet_1.zip › 6.PICRUSt2/Meta_Statistic/C372089/Wilcoxon/KO_abundance.Wilcoxon.diff.top30.heatmap.pdf]

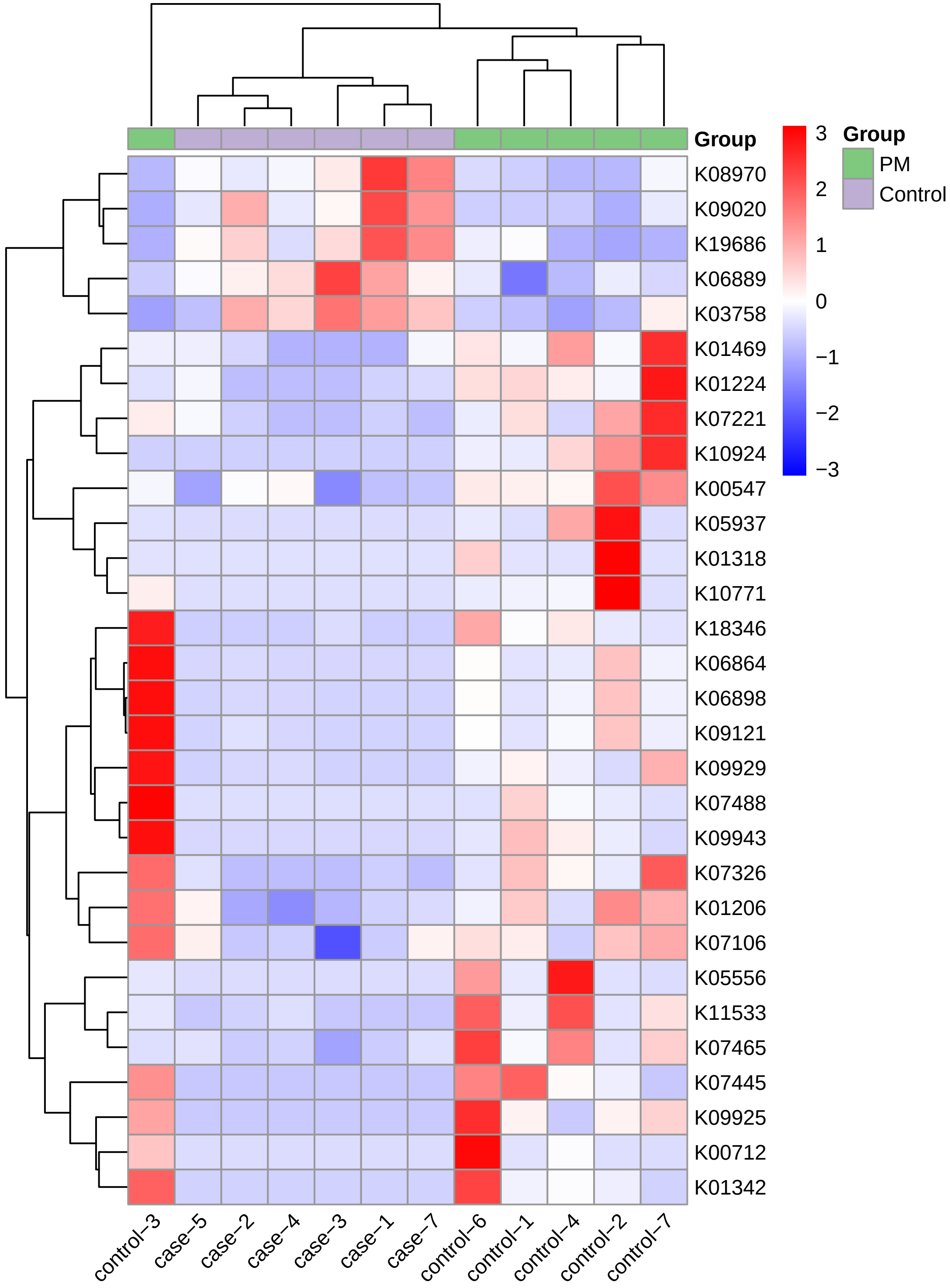
Group
K08970
K09020
K19686
K06889
K03758
K01469
K01224
K07221
K10924
K00547
K05937
K01318
K10771
K18346
K06864
K06898
K09121
K09929
K07488
K09943
K07326
K01206
K07106
K05556
K11533
K07465
K07445
K09925
K00712
K01342
3
2
1
0
−1
−2
−3
Group
PM
Control
control−3
case−5
case−2
case−4
case−3
case−1
case−7
control−6
control−1
control−4
control−2
control−7
